# Expression Profiling in *Bemisia tabaci* under Insecticide Treatment: Indicating the Necessity for Custom Reference Gene Selection

**DOI:** 10.1371/journal.pone.0087514

**Published:** 2014-01-31

**Authors:** Pei Liang, Yajie Guo, Xuguo Zhou, Xiwu Gao

**Affiliations:** 1 Department of Entomology, China Agricultural University, Beijing, P. R. China; 2 Department of Entomology, University of Kentucky, Lexington, Kentucky, United States of America; Ghent University, Belgium

## Abstract

Finding a suitable reference gene is the key for qRT-PCR analysis. However, none of the reference gene discovered thus far can be utilized universally under various biotic and abiotic experimental conditions. In this study, we further examine the stability of candidate reference genes under a single abiotic factor, insecticide treatment. After being exposed to eight commercially available insecticides, which belong to five different classes, the expression profiles of eight housekeeping genes in the sweetpotato whitefly, *Bemisia tabaci*, one of the most invasive and destructive pests in the world, were investigated using qRT-PCR analysis. In summary, e*longation factor 1α* (*EF1α*), *α-tubulin* (*TUB1α*) and *glyceraldehyde-3-phosphate dehydrogenase* (*GAPDH*) were identified as the most stable reference genes under the insecticide treatment. The initial assessment of candidate reference genes was further validated with the expression of two target genes, a P450 (*Cyp6cm1*) and a glutathione *S*-transferase (*GST*). However, ranking of reference genes varied substantially among intra- and inter-classes of insecticides. These combined data strongly suggested the necessity of conducting custom reference gene selection designed for each and every experimental condition, even when examining the same abiotic or biotic factor.

## Introduction

Quantitative real-time reverse transcription polymerase chain reaction (qRT-PCR) is one of the most effective and sensitive techniques for gene expression analysis [1]–[4]. However, enabling comparisons across different samples, qRT-PCR data must be normalized to correct variations in pipetting, RNA concentration, reverse-transcription, and efficiency of PCR amplification [2], [5], [6]. The most common normalization method is to compare the mRNA level of the target gene with that of a reference gene whose expression level is considered stable regardless of different experimental conditions [7]–[9]. However, none of the reference genes discovered thus far is consistently expressed in a universal and invariant way under various experimental conditions [10], [11], and recent reference gene selection studies indicate that a single reference gene is generally insufficient to normalize the expression data of all target genes [12]–[16].

In insect, the reference genes have been validated at least in the desert locust *Schistocerca gregaria* [17], the oriental fruit fly *Bactrocera dorsalis* [18], the fruit fly *Drosophila melanogaster* [19] emerald ash borer *Agrilus planipennis* [20], the diamondback moth *Plutella xylostella* [21], the tobacco whitefly *Bemisia tabaci* [22], and the red imported fire ant, *Solenopsis invicta* [23] under a diverse set of biotic and abiotic conditions. However, no one single universal reference was identified, either. Therefore, it is not surprising that no single universal reference is available for four different lepidopteran insect species [24]. In this context, reliable reference genes for gene expression analysis based on different experimental conditions should be selected.

The tobacco whitefly, *B. tabaci*, is an invasive insect pest of agriculture and horticulture worldwide [25]. Because of the application of chemical insecticides has been the primary strategy for the control of *B. tabaci*, this pest has developed different levels of resistance to a wide range of insecticides [26]–[33]. It has been well documented that insecticide resistance in *B. tabaci* usually is associated with enhanced detoxification by oxidative and hydrolytic pathways [34]–[36]. Therefore, increasing numbers of studies are using the RT-qPCR techniques to detect the changes of mRNA expression of detoxifying enzymes genes in resistant populations and tried to provide new insights into insecticides resistance mechanisms [37], [38].

Though the suitable references genes has been documented in bacterially challenged bees [39], in *Tribolium* beetles infected with fungus [40], in plant virus infected *B. tabaci* [22] and in Bt toxin treated *P. xylostella* [21], this kind of information is lacking for insects stressed by different types of chemical insecticides.

In this study, a set of reliable reference genes for gene expression analysis in the *B. tabaci* biotype Q, one of the most invasive and destructive pest in the world, after exposure to eight commonly used insecticides (which belong to five different classes) was selected and then valuated with two target genes, a P450 gene (*Cyp6cm1*) and glutathione *S*-transferase gene (*GST*). Over expression of the P450 has been proved to be responsible for neonicotinoid insecticides resistance in *B. tabaci* [35], [37] while this is not the case for GST. The objective of this work is to provide a set of universal reliable reference genes for research of genes with toxicological function in *B. tabaci*.

## Materials and Methods

### Ethics Statement

*Bemisia tabaci* biotype Q strains used in this study were initially collected in the field at Beijing in 2010, and have been maintained in in our laboratory at the China Agricultural University for three years without exposure to any insecticide. No specific permit was required for the described field collections, and the location is not privately-owned or protected in any way. The species in the genus of *Bemisia* are common agricultural pests and are not included in the “List of Endangered and Protected Animals in China”.

### Leaf-dip bioassay and *B. tabaci* susceptibility to various insecticides

A total of eight kinds of insecticides commonly been used in the management of *B. tabaci* were used in this study, including chlopyrifos, beta-cypermethrin, carbosulfan, abamectin, buprofezin and three neonicotinoids (imidacloprid, acetamiprid, nitenpyram. All insecticides used were of technical grade with purity greater than 95%.

First, the LC_50_ of each insecticide to *Bemisia tabaci* biotype Q was determined using the leaf-dip method [41]. Briefly, the stock solution of insecticide was diluted to five to seven concentrations with 0.02% Triton X-100, then eggplant leaf discs (33 mm in diameter) were dipped in aqueous solutions of insecticides for 10 s After being air-dried for 1 h, the leaf disc was placed abaxial side down on the bed of agar (2%) within the plug seal cap of a 100 ml centrifuge tube (the bottom of the tube was cut off and covered with a piece of black cotton cloth, the tube was 8 cm in height and 3.8 cm in diameter). Approximately 30–50 adult whiteflies were transferred into the tubes and the tubes were thereafter covered with the caps (with leaf disc inside). Adult whiteflies treated with 0.02% Triton X-100 were used as control. Four replicates of each concentration were carried out. The adults were allowed to feed on the treated disc for 48 h or 72 h (depending on classes of insecticide) at 25±2°C, 75% RH, and a 16∶8-h (light∶dark) photoperiod. LC_50_ values and their virulence regression equation slope were calculated using PoloPlus™ software (LeOra Software, Berkeley, USA). The bioassay results were listed in Table S1.

And then the similar leaf-dip method was used for treatment of *B. tabaci* except that the eggplant leaf discs were dipped at a concentration corresponding to the LC_50_ of each insecticide, respectively. About 300 adults were treated for each insecticide. The adults were allowed to feed on the treated disc for 48 h at 25±2°C, 50–70% RH, and a 12∶12 h (light∶ dark) photoperiod. Adult whiteflies treated with 0.02% Triton X-100 were used as control. The surviving insects were collected for subsequent RNA extraction. Three biological replicates were performed for each insecticide treatment.

### Total RNA extraction and cDNA synthesis

Total RNA was extracted from 100 to150 *B. tabaci* adults using Trizol Reagen (Invitrogen, Carlsbad, CA) following the manufacturer's instructions. RNA concentration and quality were measured according to the optical density at 260 nm and the A260/A280 absorption ratio using a NAS-99 spectrophotometer (ACTGene, USA). RNA samples with an A260/A280 ratio ranging from 1.8 to 2.0 and A260/A230>2.0 were used for analysis. All RNA samples were adjusted to the same concentration to homogenize RNA input in the subsequent reverse-transcription reaction. One microgram of RNA was reverse transcribed into first-strand cDNA using a Thermo Scientific Verso™ cDNA Synthesis Kit (Thermo Scientific, Wilmington, DE, USA). The cDNA was stored at −20°C until use.

### Primer design and quantitative real-time PCR

A total of eight candidate reference genes, including five commonly used reference genes, *β-actin* (*ACT*), α-tubulin (*TUB1α*), *elongation factor 1α* (*EF1α*), *glyceraldehyde-3-phosphate dehydrogenase* (*GAPDH*) and *18S ribosomal RNA*(*18S rRNA*), as well as three rarely used reference genes *ribosomal protein L13a* (*RPL13A*), *cyclophilin 1*(*CYP1*) and *TATA box binding protein-associated factor* (*TBP-AF*) in *B. tabaci* were chosen for valuation of their expression stability in *B. tabaci*. The sequences, length of products, and source of these candidate genes were listed in Table 1.

**Table 1:** Candidate reference genes and primers used for qRT-PCR analysis.

Gene	Molecular function	Accession No.	Primer sequences (5′ to 3′)^a^	Product length (bp)	Tm^b^ (°C)	E^c^	R^2^ d
*ACT*	β-actin	AF071908	F:ACCGCAAGATTCCATACCC R:CGCTGCCTCCACCTCATT	127	60	103.5	0.996
*TUB1α*	Tubulin alpha-1 chain	EE598061	F:CACTGTTGTTCCTGGTGGC R:AGTGGACGAAAGCACGCTTG	140	60	93.6	0.999
*EF1α*	elongation factor1-alpha	EE600682	F:GATGGCACGGAGACAATATG R:TTGTCAGTGGGTCTGCTAGG	138	60	94.5	0.995
*GAPDH*	glyceraldehyde-3-phosphate dehydrogenase	JU470454	F:AAATGACTTTCCCTACAGC R:ATTATGGCGTGATGGC	82	60	90.2	0.996
*18S rRNA*	18S ribosomal RNA	Z15051	F:CGGCTACCACATCCAAGGAA R:GCTGGAATTACCGCGGCT	112	60	92.6	0.998
*RPL13A*	ribosomal protein L13a	EE596312	F:CATTCCACTACAGAGCTCCA R:TTTCAGGTTTCGGATGGCTT	101	60	99.2	0.996
*CYP1*	cyclophilin 1	EE596217	F:CACCGTGTCATCCCCAACTT R:GTGTGCTTGAGGGTGAAGTT	118	60	88.5	0.997
*TBP-AF*	TATA box binding protein-associated factor	EE596204	F:TGTGGGACACCCATTATCAG R:TGTGCAGCCAAGGAAATAAG	162	60	96.5	0.995
*CYP6CM1*	cytochrome P450 CYP6cm1	GQ214539	F:GCCATCGGTGATAAAGGAGA R:AACTCGGTTTCCTCATCGTG	128	60	91.4	0.996
*GSTs*	Glutathione *S*-transferase gene	EU723684	F:GTGGAGGAAAAACACCCTCA R:AGTCGGTTTTTGGCCTCTTT	97	60	90.8	0.995

a : F, forward primer; R, reverse primer.

b : Tm, Melting temperature.

c : E, Efficiency.

d : R^2^, Coefficient of correlation.

Real-time PCR was conducted using an ABI 7500 Real Time PCR System (Applied Biosystems, Foster, CA) and the ROX's Platinum SYBR Green qPCR SuperMix-UDG kit (Invitrogen, Carlsbad, CA). The reactions were performed in a 20 µL mixture contained 1 µL of cDNA template, 10 µL of SYBR Green qPCR SuperMix-UDG, 1 µL of each primer and 7 µL of nuclease-free water. The optimized real-time PCR program consisted of an initial step at 50°C for 2 min, 95°C for 2 min, followed by 40 cycles of 95°C for 15 s and 60°C for 30 s. Relative standard curves for the transcripts were generated with serial dilutions of cDNA (1/5, 1/15, 1/45, 1/135, and 1/405). The corresponding qRT-PCR efficiencies (E) were calculated according to the equation: E = (10^[−1/slope]^−1)×100 [42].Three independent biological duplications were performed for all the reference genes studied and data for each biological duplicate were carried out in triplicate. The dissociation curves were obtained after amplification to inspect the specificity of the primer sets.

### Statistical analysis

The raw data of qPCR were analyzed using ABI 7500 SDS System software (version 2.0) (Applied Biosystems) and the threshold cycle (Ct value, the cycle at which the fluorescent signal was first significantly different from the background) for each gene-cDNA sample was determined automatically. All biological replicates (each contained three technical replications) were used to calculate the average Ct values using geNorm and NormFinder software packages as described in their manuals. Ct values were converted into relative quantities and imported into the *geNorm* and the *NormFinder* software for further analysis. The geNorm provides a measure of gene expression stability (M), and creates stability ranking via a stepwise exclusion of the least stable gene. Genes with the lowest M values have the most stable expression. The *geNorm* also calculates a serial values of Vn/Vn+1, which indicates the pairwise variation between two sequential normalization factors and determines the optimal number of reference genes required for accurate normalization. A value below 0.15 indicates that an additional reference gene will not significantly improve normalization. Ct values were converted into relative quantities and further analyzed by *RefFinder*, a user-friendly web-based comprehensive tool (http://www.leonxie.com/referencegene.php), to evaluate the expression stabilities of candidate reference genes. The *RefFinder* was developed for evaluating and screening reference genes from the extensive experimental datasets. It integrated the currently available major computational programs (*geNorm*, *Normfinder*, *BestKeeper*, and the *comparative ΔCt method*) [11], [43]–[45] to compare and rank the tested candidate reference genes. Based on the rankings from each program, it assigns an appropriate weight to an individual gene and calculated the geometric mean of their weights for the overall final ranking. Then starting with the most stable genes the *geNorm* program was used to calculate the pair-wise variation V of two consecutive normalization factors (NF) that result from stepwise introduction of another gene, and the generally adopted threshold of V = 0.15 was used for decision of the most reliable reference gene combination.

One-way ANOVA was used to compare the relative expression levels of selected target genes (*Cyp6cm1* and *GST*) calculated using three and more sets of optimal reference genes, and Student's *t-test* for comparison of target gene expression calculated with two sets of reference genes. Both statistical analysis were conducted using SPSS 17.0 for windows (SPSS Inc., Chicago, IL) with a significance level set at *P* = 0.05.

## Results

### Amplification specificity and efficiency of the primer sets

**Figure 1:**
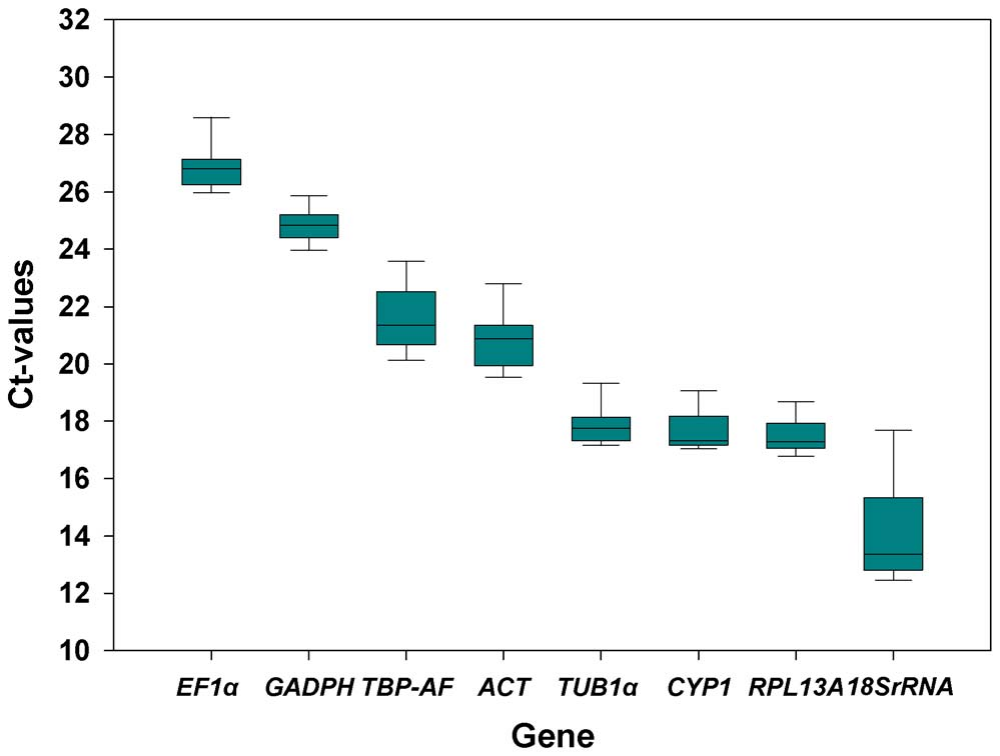
Expression levels of candidate reference genes. The expression level of candidate *Bemisia tabaci* reference genes in the tested samples is shown in terms of the cycle threshold number (Ct-value). The box plot indicates the mean of duplicate samples; the whiskers indicate the standard error of the mean.

Single peaks in the dissociation curves further demonstrated the specificity of all primer sets (data not shown). A standard curve was generated for each gene, using three-fold serial dilutions of the pooled cDNA generated from each experiment. The correlation coefficient and PCR efficiency characterizing each standard curve are given in Table 1. Box plots of raw Ct values of the candidate reference genes among samples were produced respectively, from which the expression stability of the candidate reference genes can be told intuitively (Fig. 1). The eight candidate reference genes expressed different transcription levels from each other, with Ct values spanning 14.23–26.91, in which the lowest Ct (and highest expression) corresponded to 18SrRNA and the highest Ct to *EF1α*.

### Expression stability analysis of the candidate genes and optimum number of genes for normalization

**Figure 2:**
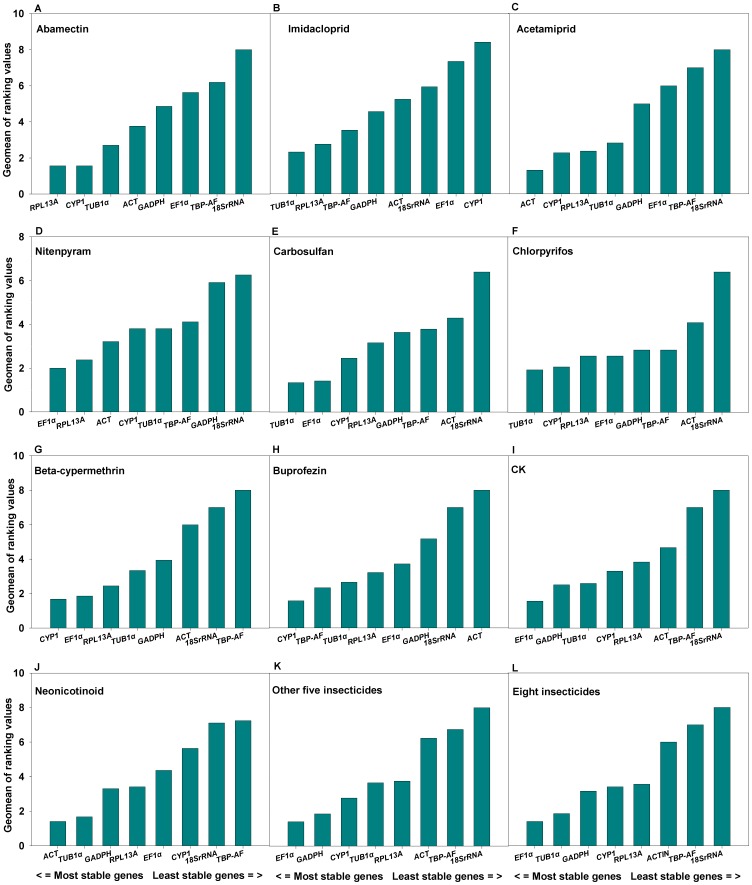
Expression stabilities of the candidate reference genes. The average expression stability of the reference genes was measured using Geomean method by *RefFinder*. A lower Geomean of ranking value indicates more stable expression. **CK**: Treated with 0.02% Triton X-100 only. **Other 5 insecticides**: treated by chlopyrifos, beta-cypermethrin, carbosulfan, abamectin and buprofezin, respectively. **Eight insecticides:** treated with each of eight insecticides used in this work.

**Figure 3:**
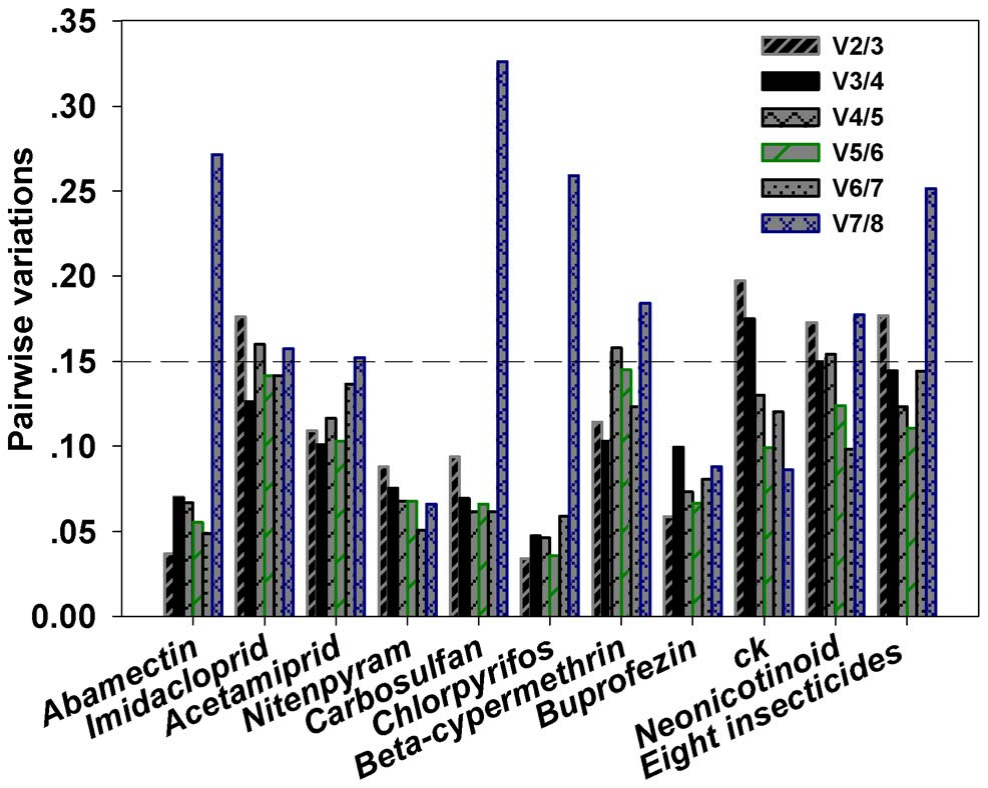
Optimal number of reference genes for normalization in *Bemisia tabaci*. Average pairwise variations (V) were calculated in *geNorm* between the normalization factors NFn and NFn+1 to indicate whether inclusion of an extra reference gene would add to the stability of the normalization factor. The value of Vn/Vn+1 indicates the pairwise variation between two sequential normalization factors and determines the optimal number of reference genes required for accurate normalization. A value below 0.15 indicates that an additional reference gene will not significantly improve normalization.

The expression profiles of the eight candidate reference genes were assessed in *B. tabaci* treated with eight different insecticides belong to five classes. For the whiteflies treated by LC_50_ of abamectin, according to the ranking of stability generated with *RefFinder*, *RPL13A* was the most stable gene with the lowest ranking value (1.8) followed by *CYP1*, *TUB1α*, *ACT*, *GADPH*, *EF1α*, *TBP-AF* and the *18SrRNA*was the most unstable one (Figure 2A). If we consider the generally adopted threshold of V = 0.15, the *geNorm* analysis revealed that two most stable genes are needed for a reliable normalization, because of the addition of a third gene does not result in any appreciable improvement of the normalization factor (Figure 3). Thus the *RPL13A* and *CYP1* were identified as the most stable pair of genes.

Similar analyses were conducted for the remaining treatments. For the whiteflies treated by LC_50_ of imidacloprid, the eight candidate reference genes were ranked (from the highest to lowest stability) by the *RefFinder* as *TUB1α<RPL13A<TBP-AF<GADPH<ACT<18SrRNA<EF1α<CYP1* (Figure 2B). And the *geNorm* analysis revealed the optimum combination of *TUB1α*, *RPL13A* and *TBP-AF* possessed the least variation in their expression ratios (Figure 3), therefore these three genes were identified as the most stable reference gene set.

For the acetamiprid treated group, the order of gene expression stability (from the most stable to the least stable) was calculated as *ACT<CYP1<RPL13A<TUB1α<GADPH<EF1α<TBP-AF<18SrRNA* by *RefFinder* (Figure 2C). And the *geNorm* analysis revealed the optimum pair of genes with least variation was *ACT* and *CYP1* (Figure 3). Therefore, the reference genes of *ACT* and *CYP1* were identified as the most stable pair of genes.

In whiteflies treated with LC_50_ of nitenpyram, the eight candidate reference genes were ranked (from the highest to lowest stability) by the *RefFinder* as *EF1α<RPL13A<ACT<CYP1<TUB1α<TBP-AF<GADPH<18SrRNA* (Figure 2D). And the results of *geNorm* analysis identified *EF1α* and *RPL13A* as the most stable reference gene pair.

For the carbosulfan treated group, according to *RefFinder*, the expression stability (from the highest to the lowest) of the eight candidate reference genes was ranked as *TUB1α<EF1α≪CYP1<RPL13A<GADPH<TBP-AF<ACT<18SrRNA* (Figure 2E). Analysis with *geNorm* (Figure 3) revealed that the *TUB1α* and *EF1α* made of the most stable gene pair.

In the chlorpyrifos treated group, the overall final order of the genes from the most stable to the least stable was: *TUB1α<CYP1<RPL13A<EF1α<GADPH<TBP-AF<ACT<18SrRNA* (Figure 2F). And the *TUB1α* and *CYP1* were identified as the most stable reference gene pair according to the V values calculated with the *GeNorm* (Figure 3).

Stabilities of the eight genes expressed in *B. tabaci* treated with beta-cypermethrin were ordered as *CYP1<EF1α<RPL13A<TUB1α<GADPH<ACT<18SrRNA<TBP-AF* (Figure 2G). Combined with the V value calculated by *GeNorm* (Figure 3), the reference genes *CYP1*and *EF1α* were considered the most reliable pair for gene expression normalization.

For the buprofezin treated group, the expression stability of the eight genes were ranked as *CYP1<TBP-AF<TUB1α<RPL13A<EF1α<GADPH<18SrRNA<ACT* by RefFinder from the highest to the lowest (Figure 2H). Considering the V value resulted from the *GeNorm* analysis *CYP1*and *TBP-AF* were identified as the most stable gene pair.

The stability order of the eight genes expressed in the control (untreated) group, from the most stable to the least stable, was lined as *EF1α<GADPH<TUB1α<CYP1<RPL13A<ACT<TBP-AF<18SrRNA* (Figure 2I). And the *GeNorm* analysis revealed that *EF1α* and *GADPH* was the optimum gene pair for a reliable normalization.

When the imidcloprid, acetamiprid and nitenpyram treated group was combined as neonicotinoid treated goup, the stability of the eight candidate genes (from the highest to the lowest) was ranked as *ACT<TUB1α<GADPH<RPL13A<EF1α<CYP1<18SrRNA<TBP-AF* (Figure 2J). And according to the V values, *ACT*, *TUB1α* and *GADPH* was considered as the most stable reference gene set for normalization.

Combining all the five non-neonictinoid insecticides together for consideration, the *RefFinder* ranked the eight tested gene from the most stable to the least stable as *EF1α<GADPH<CYP1<TUB1α<RPL13A<ACT<TBP-AF<18SrRNA* (Figure 2K). And further analysis by the *GeNorm* identified *EF1α*, *GADPH* and *CYP1* together as a reliable reference gene set.

Finally, all whitefly groups treated with eight insecticides were analyzed together and designated as the comprehensive group. According to *RefFinder*, from the most to the least stable reference genes, the overall ranking of the eight candidates across different developmental stages was: *EF1α<TUB1α<GADPH<CYP1<RPL13A<ACT<TBP-AF<18SrRNA* (Figure 2L). As a result, *EF1α*, *TUB1α* and *GADPH* were considered as the most stable reference genes for qRT-PCR normalization (Table 2).

**Table 2:** Selected reference genes under different insecticide treatments.

Insecticide class	Insecticide	Reference genes
Avermectins	Abamectin	*RPL13A and CYP1*
Neonecotinoids	Imidacloprid	*TUB1α*, *RPL13A and TBP-AF*
	Acetamiprid	*ACT and CYP1*
	Nitenpyram	*EF1α and RPL13A*
Carbamates	Carbosulfan	*TUB1α and EF1α*
Organophosphates	Chlorpyrifos	*TUB1α and CYP1*
Pyrethroids	Beta-cypermethrin	*CYP1and EF1α*
Chitin synthesis inhibitors	Buprofezin	*CYP1*and *TBP-AF*
	Neonicotinoid	*ACT*, *TUB1α and GADPH*
	Comprehensive	*EF1α*, *TUB1α* and *GADPH*

### Validation of selected reference genes in *B. tabaci*

**Figure 4:**
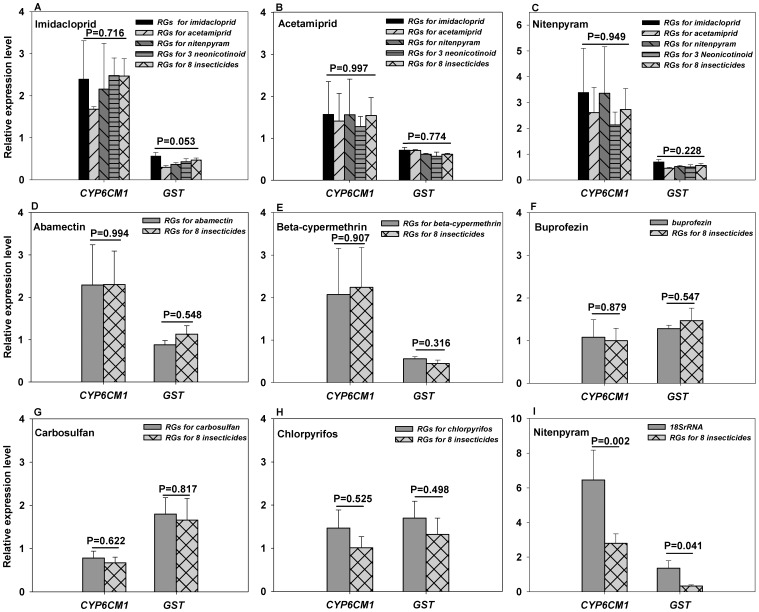
Relative expressions of target genes calculated using different sets of reference genes. The relative expression level of the two target genes were calculated according to the 2^−ΔΔCt^ method (Pfaffl 2001) and using untreated group as control. **RG:** reference gene.

The relative expression levels of two target genes, *Cyp6CM1*and *GST*, were analyzed after whiteflies treated with three neonicotinoid insecticides. No significant differences were found among the expressions of *Cyp6CM1*and *GST* using five different sets of reference genes (*P*>0.05) (Fig. 4 A–C). Similarly, when normalized with their respective optimal reference genes for each of the five non-neonicotinoid insecticides, expression levels of *Cyp6CM1*and *GST* also exhibited no apparent differences (*P*>0.05) (Table 2; Fig. 4 D–H). However, significant expression differences were found between the most stable reference genes for all eight insecticides, *EF1α*, *TUB1α* and *GADPH*, and *18S rRNA*, the optimal reference gene recommended previously Fig. 4 I, [22].

## Discussion

There exists no doubt that the qRT-PCR technology has made the quantitative determination of gene expression more convenient than ever before. However, extreme care must be taken for the selection of internal reference genes before their application of qRT-PCR. It has been documented that the selection of suitable reference genes is very important to obtain reliable and accurate data [2], [3], [46]. Currently, however, many common reference genes are used as internal controls without any evaluation of their variance and instability, and most publications only use a single internal control for normalization. These un-validated single reference genes, however, were proved to be not always reliable under various experimental conditions [12]–[16]. Therefore, more and more biologists pay their attention to the selection and validation of reliable reference gene(s) expressed stably regardless of different experimental conditions from the species they are interested in, in order to avoid unnecessary errors in qRT-PCR analysis.

Insecticide resistance is becoming a serious barrier for the sustainable control of pest insects. And the identification of insecticide resistance mechanisms would provide ways of detection and management of resistance [47]. The qPCR has been extensively used to uncover the mechanisms of insecticide resistance, and it has been proven by a lot of publications that the overexpression of detoxifying enzymes and the reduced expression of insecticide target genes were responsible for insecticide resistance [48]–[51]. Up to date, however, no universal and reliable reference genes were selected and evaluated in insects stressed with different classes of insecticides.

In the present work, a total of eight candidate reference genes were validated in the tobacco whitefly treated with eight commonly used insecticides for the control of this pest.

According to the final ranking order calculated by *RefFinder*, the five most stable reference genes for the eight tested insecticides treated whitefly were selected (Fig. 2A–H). Even for imidacloprid, acetamiprid and nitenpyram which belong to the same class of insecticides, the most stable reference genes were also different from each other (Fig. 2B–D). These results further proved that no single universal reference is available under different experiment conditions, and made the stability evaluation of reference gene necessary prior to the quantification of gene expression by qPCR.

Very interestingly, the relative expression level of two detoxifying enzymes, *Cyp6cm1* and *GST* from each of eight insecticides treated *B. tabaci* groups showed no significant difference between calculations using the selected reference gene set for each insecticide and calculations using the selected reference gene set for all eight insecticides (*EF1α*, *TUB1α* and *GADPH*). Combined with the results of Li *et al* [22] that the expression of *EF1α* and *GADPH* was stable between the thiamethoxam (a neonicotinoid insecticide) susceptible and resistant *B. tabaci* strains, we recommended that our selected reference gene set (*EF1α*, *TUB1α* and *GADPH*) can be used as reliable internal reference for the data normalization in qRT-PCR experiments using *B. tabaci* treated with different insecticides.

Li et al [22] suggested that *18SrRNA* was stably expressed in *B. tabaci* when treated with thiamethoxam or under different temperatures (4.0, 25.0, and 37.5°C). Based on the overall ranking by *RefFinder*, however, it was identified as the least stable reference gene in our study. When normalized with *18SrRNA*, expressions of *Cyp6cm1* and *GST* in *B. tabaci* treated with nitenpyram were significantly higher than those normalized with the set of most stable reference genes (Fig. 4I). These combined data strongly suggested the necessity of conducting customized reference gene selection for each and every experimental condition.

## Supporting Information

Table S1 **Toxicity of eight insecticides to adults of** ***Bemisia tabaci*** **Mediterranean.**
(DOC)
