# Supplementary material for: Expression Profiling in Bemisia tabaci under Insecticide Treatment: Indicating the Necessity for Custom Reference Gene Selection
Source: PLoS One. 2014 Jan 31;9(1):e87514. doi: 10.1371/journal.pone.0087514 (PMC3909111; doi:10.1371/journal.pone.0087514)
Supplement: Table S1 — Toxicity of eight insecticides to adults of Bemisia tabaci Mediterranean. (DOC) [file pone.0087514.s001.doc]

Table S1 Toxicity of eight insecticides to adults of *Bemisia tabaci* Mediterranean

| Insecticide class | Insecticide varieties | LC_50_ mg/l | CI_95_ | *χ*^2^ (*df* ***^a^***) | Slope ± SEM |
| --- | --- | --- | --- | --- | --- |
| Neonicotinoid | Nitenpyram | 13.1 | 9.3-16.1 | 29.72(22) | 2.71±0.10 |
|  | Imidacloprid | 747.0 | 553.0-1059.0 | 26.45(18) | 1.46±0.11 |
|  | Acetamiprid | 130.0 | 116.0-147.0 | 28.08(22) | 2.43±0.18 |
| Avermectin | Abamectin | 0.41 | 0.35-0.47 | 32.24(22) | 2.35±0.13 |
| Organophosphate | Chlorpyrifos | 2119.1 | 1831.2-2447.6 | 31.92(20) | 1.63±0.11 |
| Pyrethroid | Beta-cypermethrin | 949.2 | 773.7-1154.5 | 39.54(22) | 1.94±0.10 |
| Carbamates | Carbosulfan | 917.4 | 743.2-1058.5 | 36.24(22) | 1.63±0.12 |
| Chitin synthesis inhibitors | Buprofezin | 1725.7 | 1531.2-2057.6 | 38.32(22) | 1.84±0.13 |

a:Digree of freedom
